# Distinct relaxation mechanism at room temperature in metallic glass

**DOI:** 10.1038/s41467-023-36300-x

**Published:** 2023-02-01

**Authors:** Yi-Tao Sun, Rui Zhao, Da-Wei Ding, Yan-Hui Liu, Hai-Yang Bai, Mao-Zhi Li, Wei-Hua Wang

**Affiliations:** 1 Institute of Physics, Chinese Academy of Sciences, 100190 Beijing, China; 2 Center of Materials Science and Optoelectronics Engineering, University of Chinese Academy of Sciences, Beijing, 100049 China; 3 Songshan Lake Materials Laboratory, Dongguan Guangdong, 523808 China; 4 Department of Physics, Beijing Key Laboratory of Opto-electronic Functional Materials & Micro-nano Devices, Renmin University of China, Beijing, 100872 China

**Keywords:** Glasses, Structure of solids and liquids

## Abstract

How glasses relax at room temperature is still a great challenge for both experimental and simulation studies due to the extremely long relaxation time-scale. Here, by employing a modified molecular dynamics simulation technique, we extend the quantitative measurement of relaxation process of metallic glasses to room temperature. Both energy relaxation and dynamics, at low temperatures, follow a stretched exponential decay with a characteristic stretching exponent *β* = 3/7, which is distinct from that of supercooled liquid. Such aging dynamics originates from the release of energy, an intrinsic nature of out-of-equilibrium system, and manifests itself as the elimination of defects through localized atomic strains. This finding is also supported by long-time stress-relaxation experiments of various metallic glasses, confirming its validity and universality. Here, we show that the distinct relaxation mechanism can be regarded as a direct indicator of glass transition from a dynamic perspective.

## Abstract (web summary)

The mechanism governing structural relaxation in metallic glasses remains elusive, hampering their stability and engineering applications. Here, the authors reveal a distinct relaxation mechanism with a stretching exponent of 3/7, providing new insight for understanding the nature of glass.

## Introduction

Glass is inherently out of equilibrium and undergoes structural relaxation towards states of lower energy, but with extremely sluggish dynamics usually described as “frozen” below glass transition temperature (*T*_*g*_)^1–5^. However, the stability of a glass and its engineering applications are affected by the extremely slow structural relaxation^6^, meanwhile the mechanism that governs structural relaxation at low temperatures is still elusive, mainly due to its difficult measurements in either experiments or numerical simulations.

Structural relaxation in glass-forming liquids above *T*_*g*_ has been massively investigated through experiments and numerical simulations, where rich dynamics were revealed^7^. At temperatures well above melting point, the structural relaxation of a liquid melt usually follows a simple exponential decay. As the liquid melt approaches supercooled liquid states, the structural relaxation is no longer described by a simple exponential, but takes the form of stretched exponential decay, which can be described by Kohlrausch–Williams–Watts (KWW) function of ${{\exp }}[{-(t/\tau )}^{\beta }]$, with *τ* and *β* being the relaxation time and shape parameter (stretching exponent), respectively^8,9^. With temperature decreasing, the relaxation time *τ* of supercooled liquids increases drastically, while *β* remains less than 1. Below a critical temperature, the relaxation time becomes infinite, as predicted by mode-coupling theory^10^, indicating that the relaxation time in glasses is far beyond the directly accessible experimental time scales. As demonstrated by an industrial silicate glass of Corning Gorilla Glass, it took over 1.5 years to obtain measurable dimensional changes of ~10 ppm linear strain at room temperature^11^. Thus, the mechanism of structural relaxation at low temperatures remains hidden, yet the difference in dynamics between glassy solids and glass-forming liquids might be the key to the mystery of glass^12^.

It is commonly believed that the stretching exponent *β* reveals the characteristic of structural relaxation dynamics^13,14^. In the experimental measurements of relaxation in Gorilla Glass at room temperature, the relaxation was found to follow stretched exponential decay with the value of *β* = 3/7^11^. However, a stretching exponent *β* = 3/5 was found in the potential energy relaxation of realistic alkali aluminosilicate glasses at temperature far below *T*_*g*_ based on molecular dynamics (MD) simulations^15^. Recently, many experiments focused on metallic glasses (MGs), because of the much faster relaxation dynamics compared to traditional silicate glasses. By measuring crystallization processes^16^, elasticity properties^17^, stress relaxations^18^, and structural relaxation dynamics^19^ in MGs, some non-trivial aging dynamics at or near room temperature have been revealed. However, various values of stretching exponent *β* were also found. The stress relaxation of CuZrAl MGs below *T*_*g*_ shows $\beta \sim 0.4-0.5$, while viscosity equilibration or aging for supercooled metallic liquids finds $\beta \sim 0.7-0.9$, indicating fundamentally different relaxation mechanisms in supercooled and glassy states^19,20^. Thus, the value of *β* indicates different structural relaxation mechanisms in supercooled liquids and glasses. Exploring the change of *β* in glass transition may provide deeper insights into the physical origin of structural relaxation.

In this work, we apply a vibration-accelerated aging technique based on MD simulations, so that the structural relaxation in Zr_70_Cu_30_ MG at low temperatures, both around and far below *T*_*g*_, can be systematically measured and explored in directly accessible simulation time scales. The evolution of potential energy for as quenched Zr_70_Cu_30_ MG follows a stretched exponential decay with characteristic stretching exponent $\beta=3/7$. below *T*_*g*_, while at 700 K above *T*_*g*_ the potential energy decay is better fitted with $\beta=3/5$. Stress-relaxation experiments for various MGs at different temperatures below *T*_*g*_ also find that the stress relaxation follows stretched exponential decay with $\beta=3/7$ We have explained the distinct relaxation mechanisms that differentiates glass and glass forming liquids.

## Results

### Distinct relaxation dynamics in metallic glasses below *T*_*g*_

**Fig. 1:**
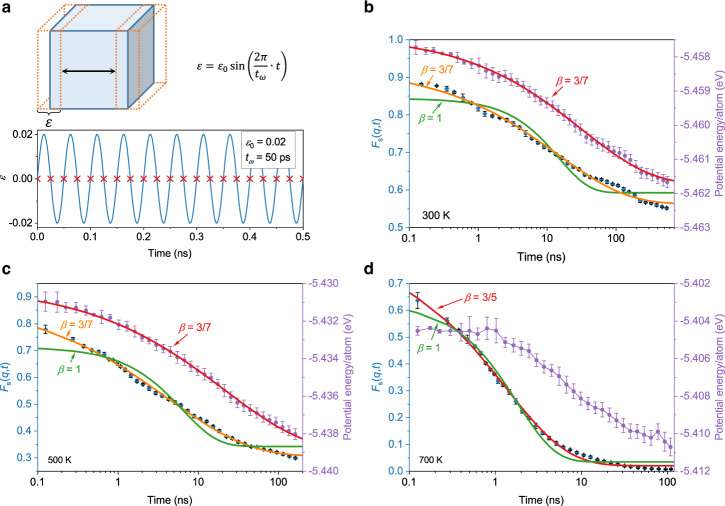
Vibration-accelerated aging and distinct relaxation dynamics. **a** Schematic of the simulation procedures of vibration-accelerated aging. A sinusoidal tension-compression strain was applied along one direction with maximum strain of 0.02 and period of 50 ps. The potential energy and structural information were monitored when strain equals 0 (marked by red crosses). Self-intermediate scattering function (diamonds) and potential energy (dots) evolutions of Zr_70_Cu_30_ MGs in accelerated aging at 300 K (**b**), 500 K (**c**), and 700 K (**d**), respectively. Solid curves are the fittings of SISF and potential energy by KWW function with $\beta=3/7$ and $\beta=1$ in **b** and **c**, and with $\beta=3/5$ and $\beta=1$ in **d**, respectively. Error bars represent standard deviations.

Fig. 1a illustrates a sinusoidal tension-compression strain of $\varepsilon={\varepsilon }_{0}{{\sin }}\left(2\pi t/{t}_{\omega }\right)$ applied to MD generated samples along *x* direction (see Methods). Here the vibrational amplitude ${\varepsilon }_{0}=0.02$ and frequency ${t}_{\omega }=50$ ps were chosen, respectively. To analyze the structural, dynamical and energy evolution in aging process, the potential energy and atomic structural information were monitored at $\varepsilon=0$ marked with red crosses in Fig. 1a.

Fig. 1b–d show the potential energy evolution of Zr_70_Cu_30_ MG at 300 K, 500 K, and 700 K in the accelerated aging process for more than 200 ns, respectively. It can be seen that potential energy decreases continuously in aging process. To further investigate the dynamical evolution of Zr_70_Cu_30_ MG in the accelerated aging process, we analyzed the self-intermediate scattering functions (SISFs) ${F}_{s}\left(q,t\right)$ (see methods) at 300 K, 500 K, and 700 K shown in Fig. 1b–d, respectively, which exhibit non-exponential decay behavior. To understand the decay behavior of potential energy and SISF in aging process at different temperatures, we fitted these decay curves by KWW function as shown in Fig. 1b–d. By comparing the decay of potential energy and SISF at 300 K, 500 K, and 700 K, three major differences are revealed. Firstly, at temperatures below *T*_*g*_, *i.e*., 300 K and 500 K, the decay of SISFs follows stretched exponential function with $\beta=3/7$, whereas at 700 K (above *T*_*g*_) the stretching exponent is found to be $\beta=3/5$. Secondly, ${F}_{s}\left(q,t\right)$ below *T*_*g*_ does not decay down to zero in our simulation time scale, indicating that a certain portion of atoms undergoes very limited displacements in the whole aging process. The third difference is that the decay of potential energy goes roughly synchronized with the decay in ${F}_{s}\left(q,t\right)$ below *T*_*g*_, indicating that the change of potential energy is a direct result of atomic rearrangements. However, the significant decay of potential energy at 700 K occurs after ~1 ns, in contrast to SISF, indicating that above *T*_*g*_ the relaxation dynamics is unnecessarily accompanied by the decay of potential energy. Thus, the relaxation dynamic in MG below *T*_*g*_ is essentially an aging dynamic that shows distinct behavior, compared to that above *T*_*g*_.

**Fig. 2:**
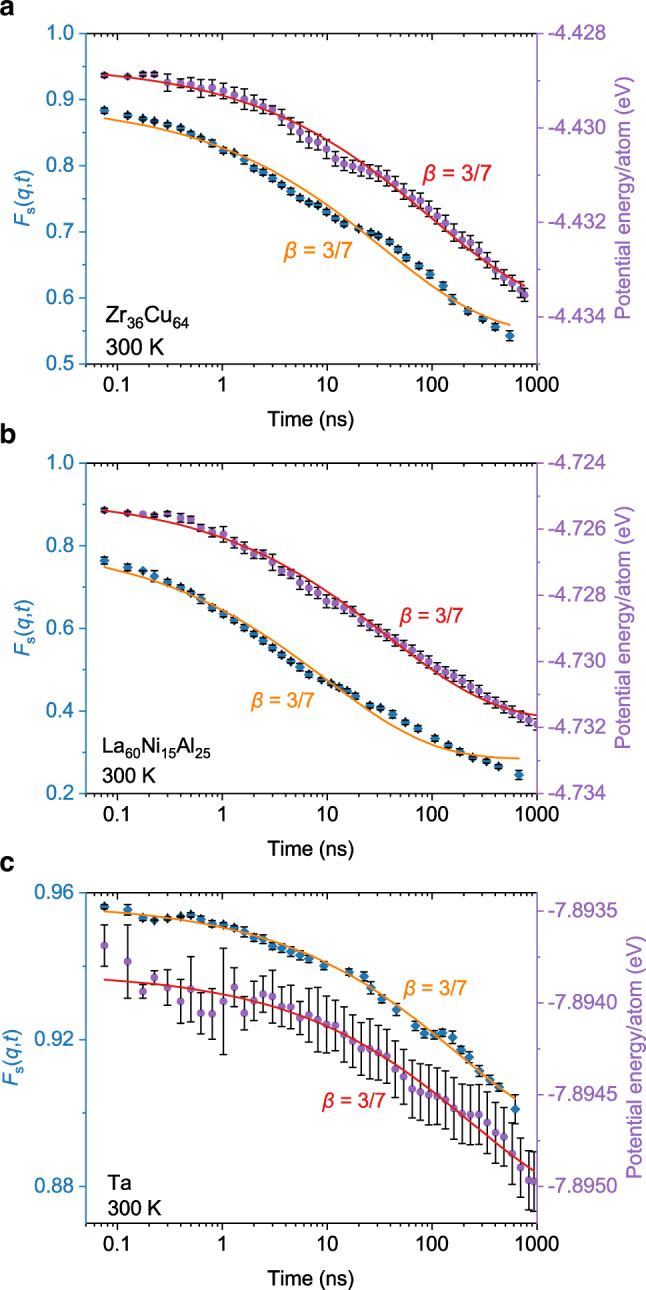
Room temperature relaxation dynamics for various metallic glasses. **a-c** Self-intermediate scattering function (diamonds) and potential energy (dots) evolutions of Zr_36_Cu_64_ (**a**), La_60_Ni_15_Al_25_ **(b)**, and Ta (**c**) in the vibration-accelerated aging at 300 K, together with fittings (solid lines) of KWW function with $\beta=3/7$. Fitting results indicate that aging dynamics in various MGs at room temperature follows a universal stretched exponential decay with $\beta=3/7$. Error bars represent standard deviations.

To check whether the aging dynamics with stretching parameter of $\beta=3/7$ below *T*_*g*_ is a general feature in MGs or not, we also performed the same vibration-accelerated MD simulations for binary Zr_36_Cu_64_ MG^21^, ternary La_60_Ni_15_Al_25_ MG^22^, and monoatomic Ta MG^23^ at room temperature up to 1000 ns, respectively. Fig. 2 shows the decay of SISF and potential energy in three types of MGs at 300 K as well as the fitting of KWW function, respectively. It is clearly seen that all decay curves can be well fitted by KWW function with $\beta=3/7$, consistent with the results shown in Fig. 1b–d. This firmly indicates that aging dynamics in various MGs at room temperature follows a universal stretched exponential decay with $\beta=3/7$, independent of composition and element.

Note that we tested the effect of the strain amplitude and frequency in the applied sinusoidal strain on the stretching exponent in aging dynamics (see Supplementary Fig. 1). It is found that frequency affects very little the decay of potential energy or SISF, while the strain amplitude has more significant effects. Larger strain amplitude induces faster aging of MGs below *T*_*g*_. However, different strain amplitude or frequency does not change the aging behavior, with all data being well fitted by KWW function with $\beta=3/7$. This indicates that the observed aging dynamics with $\beta=3/7$ is independent of strain amplitude and frequency in the vibration-accelerated MD simulations. Note that to observe the stretched exponential decay with $\beta=3/7$ in potential energy or SISF, the initial sample should be in a hyperquenched state. We tested accelerated aging in samples with different waiting times, and found larger values of $\beta$ as waiting time increases, which is the nature of stretched exponential decay, confirmed by the fitting result of standard KWW functions (see Supplementary Figs. 2 and 3).

### Stress-relaxation experiments of various metallic glasses

**Fig. 3:**
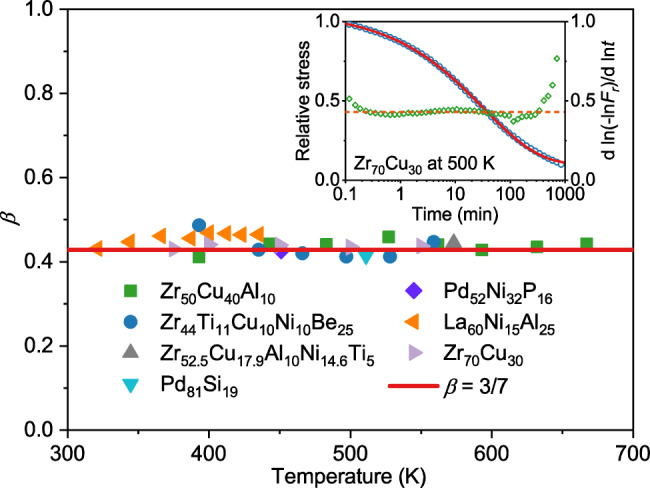
Stress-relaxation of various metallic glasses. $\beta$ values in various MGs at temperatures below *T*_*g*_ obtained by fitting the stress relaxation curves measured in experiments. The solid horizontal line (red) marks $\beta=3/7$. Inset shows a typical stress relaxation of Zr_70_Cu_30_ MG ribbon at 500 K measured in experiments with a strain of 0.005 applied to MG samples. Red solid curve is the fitting by KWW function with $\beta=3/7$. ${{{{{\rm{d}}}}}}{{{{{\rm{ln}}}}}}\left(-{{{{{\rm{ln}}}}}}{F}_{r}\right)/{{{{{\rm{d}}}}}}{{{{{\rm{ln}}}}}}t$ is also shown in the inset with the value close to 3/7. Results suggest that the$\,\beta=3/7$ dynamic signature is a common feature in physical aging below *T*_*g*_ for hyperquenched MGs.

As shown above, a distinct room temperature aging with $\beta=3/7$ is well observed in various modeled MGs by vibration-accelerated aging in MD simulations. To confirm the validity and universality of the distinct aging dynamics, we further performed stress-relaxation experiments covering thousands of minutes on different MGs at different temperatures below *T*_*g*_ (see Methods). Inset in Fig. 3 shows a representative of the stress-relaxation curve of Zr_70_Cu_30_ MG at 500 K normalized by the initial value at *t* = 0 (see Supplementary Figs. 4 and 5 for more results). It can be seen that the stress continuously decays, showing the relaxation dynamics in the aging process in MGs. Moreover, the stress decay curve can be well fitted by KWW function with $\beta=3/7$, which is consistent with the above simulation results. Various MGs exhibit similar stress relaxation behavior. More importantly, all experimentally measured stress decay curves can be well-fitted by KWW function with $\beta=3/7$. It is also found that the stress relaxation becomes slower with decreasing temperature (see Supplementary Fig. 4). Fig. 3 summarizes the values of $\beta$ in various MGs at temperatures below *T*_*g*_. The values of $\beta$ in all measured MGs are quite close to 3/7, indicating a universal mechanism that governs aging dynamics below *T*_*g*_. As shown in Fig. 3, for Zr_50_Cu_40_Al_10_ MG in a wide temperature range between 0.55 *T*_*g*_ and 0.95 *T*_*g*_, the stress relaxation process of hyperquenched MGs follows the same stretched exponential decay with $\beta=3/7$. For La_60_Ni_15_Al_25_ MG the relaxation temperature even reaches as low as 321 K, very close to room temperature. To further confirm the validity of the $\beta=3/7$ dynamic, ${{{{{\rm{d}}}}}}{{{{{\rm{ln}}}}}}\left(-{{{{{\rm{ln}}}}}}{F}_{N}\right)/{{{{{\rm{d}}}}}}{{{{{\rm{ln}}}}}}t$ was calculated after relative stress is normalized to *F*_*N*_ so that *F*_*N*_ decays from 1 to 0 at each temperature, which also reveals the stretching exponent of the decay. We can see that the value of ${{{{{\rm{d}}}}}}{{{{{\rm{ln}}}}}}\left(-{{{{{\rm{ln}}}}}}{F}_{r}\right)/{{{{{\rm{d}}}}}}{{{{{\rm{ln}}}}}}t$ straddles around 3/7 as shown in the inset in Fig. 3 (see Supplementary Fig. 6). With the stress-relaxation results, it can be concluded that the$\,\beta=3/7$ dynamic signature is a common feature in physical aging below *T*_*g*_ for hyperquenched MGs.

### Structural evolution underlying the distinct aging dynamics

To understand the distinct aging dynamics of with $\beta=3/7$ from the structural perspective, we analyzed the structural evolution of the MD-modeled Zr_70_Cu_30_ MG. We compared the radial distribution functions in the MG samples before and after 579 ns of accelerated aging. However, no significant change is observed (see Supplementary Fig. 7). Although the potential energy of the glass sample undergoes continuous decay, the overall structure of glass remains quite similar. The atomic structural evolution in aging process based on Voronoi polyhedron analysis does not show clear correlation to the decay of energy or SISF, either (see Supplementary Fig. 8). This indicates that the structural evolution in local atomic scale may not be responsible for the underlying structural basis of the observed physical aging with $\beta=3/7$.

**Fig. 4:**
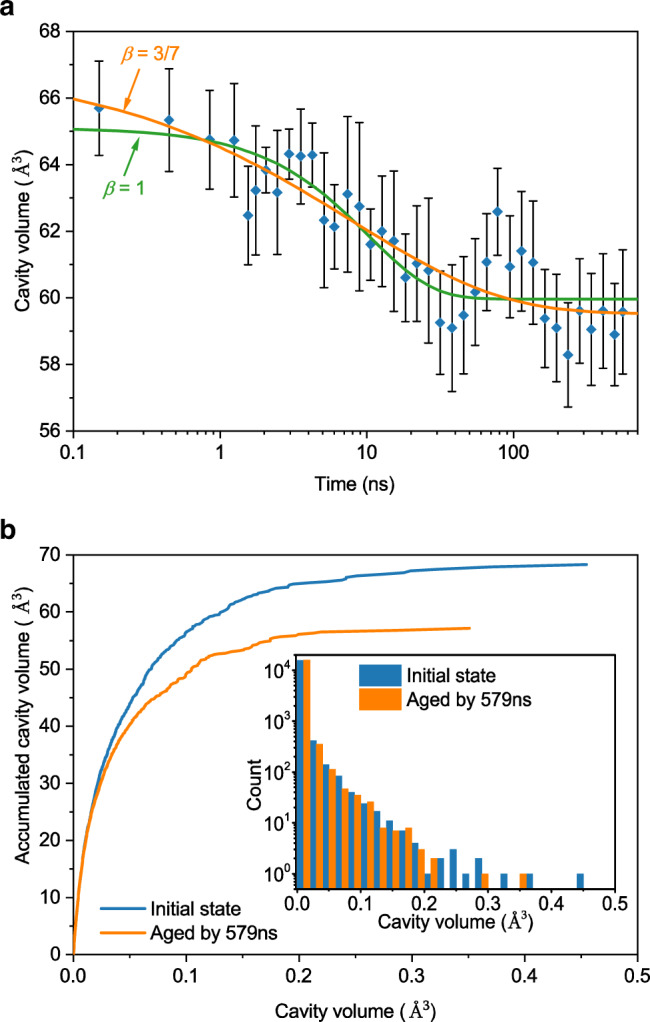
Structural evolution during accelerated aging. **a** Total cavity volume decay in MD modeled Zr_70_Cu_30_ MG in accelerated aging at 300 K. The solid orange and green curves show the fitting of KWW function with $\beta=3/7$ and $\beta=1$, respectively. Error bars represent standard deviations. **b** Accumulated distribution of cavity volume with respect to cavity volume before and after aging. Inset shows cavity volume distribution. The evolution of cavity volume may be responsible for the underlying structural basis of physical aging with $\beta=3/7$ in MGs.

To further characterize the structural difference between the samples before and after accelerated aging, we analyzed the cavities in atomic structures by the algorithms proposed by Sastry et al. (see Methods), which intrinsically exist in MGs^24^. The evolution of the total cavity volume in the modeled MGs during accelerated aging is shown in Fig. 4a. In contrast to the evolution of Voronoi polyhedral and local atomic symmetry (see Supplementary Fig. 8), cavity volume decreases in aging process, in despite of some fluctuation. More importantly, the decay of cavity volume is found to roughly follow KWW function with $\beta=3/7$, which is consistent with the decay of potential energy and SISF below *T*_*g*_. This indicates that the observed physical aging with $\beta=3/7$ in MGs below *T*_*g*_ strongly correlates to the evolution of cavity volume, which may be responsible for its underlying structural basis.

A detailed cavity analysis of samples before and after accelerated aging is shown in Fig. 4b. The major difference in the volume distribution of cavities lies in the number of big cavities, whereas the number of small cavities is similar. The accumulative cavity volume shows the contribution to total cavity volume from cavities with different volumes. In Fig. 4b, one can see that although the number of small cavities is large, their contribution to total cavity volume is relatively small. The decrease in size and number of big cavities is the major structural difference. These large cavities can be regarded as seeds for excitations that contribute to the relaxation dynamics, and dissipate during physical aging. As a result of accelerated aging, the packing density of atoms for the relaxed sample is less fluctuated, consistent with previous findings^25^.

## Discussion

The stretching exponent decay with $\beta=3/7$ in physical aging was also observed in the experimental measurements of relaxation in Gorilla Glass, an aluminosilicate glass at room temperature^11^. To understand its physical origin, a trap model was applied^14,26^. In this model, excitations are assumed to be uniformly distributed and annihilated as they diffuse to traps, leading to a stretched exponential relaxation with stretching exponent $\beta={d}_{{{{{{\rm{eff}}}}}}}/\left({d}_{{{{{{\rm{eff}}}}}}}+2\right)$, where ${d}_{{{{{{\rm{eff}}}}}}}={fd}$ is the effective dimensionality of the relaxation channels with *f* being the fraction of activated relaxation channels and *d* being real dimensionality, respectively^14,26^. For viscous liquids all channels are considered activated with $f=1$, leading to $\beta=3/5$. If the relaxation channels are equipartitioned into short- and long-range contributions, $f$ corresponds to 1/2 and a fractal dimensionality of ${d}_{{{{{{\rm{eff}}}}}}}=3/2$ is obtained for the long-range relaxation pathways, leading to $\beta=3/7$ for relaxation governed by long-range interactions^11,26^. In the case of Gorilla Glass^11^, alkali ions may move within the glass network and are assumed to be the homogeneously distributed excitations and traps, as the source of room temperature aging dynamics, resulting in $\beta=3/7$. However, no such a particular source like alkali ions is presented in MGs. It has been assumed that there exist ‘defects’ in MGs that may be excited or annihilated simultaneously by external stress in experiments or vibrations in MD simulations, acting as a source of dynamics at low temperatures similar to the mixed alkali ions in Gorilla Glass^26^. In the MD simulations, a portion of atoms is found to undergo very limited non-affine displacements, which could be considered as a glass network that constraints relaxation dynamics, while cavities may play the role of defects in MGs as shown above. It is also worth noting that a recent study on the aging dynamics in ZrCu MG found that external shear would make the system’s energy landscape fractal with a fractal dimension ~1.42, which could offer an understanding of relaxation pathways from the perspective of hyperdimensional configuration space^27^.

However, such a universal stretching parameter of $\beta=3/7$ has not been reported for organic glasses in aging process in previous findings as far as we searched. For relaxation dynamics in organic glasses, previous studies mainly focused on the dielectric relaxation spectrum. By fitting the *α*-relaxation peak with KWW function, a *β* value can be also obtained, which shows a relatively large distribution in a variety of organic glasses^28,29^, instead of a universal one. Similarly, for MGs *β* values obtained by fitting of the *α*-relaxation peak in loss modulus spectrum also distribute in between 0.4~0.6^30,31^. The fundamental difference is that in loss modulus or dielectric relaxation spectrum the response of a material is measured as an electric field or external stress is applied, with the response time insignificant compared to the time-scale of aging. Hence, the effect of aging is usually neglected, and KWW function is used to obtain the distribution of elementary events that comprise *α*-relaxation. The *β* values obtained by fitting *α*-relaxation peak in loss modulus or dielectric relaxation spectrum could be different from those obtained by fitting the decay curves of SISFs or stress-relaxation measurements in our work. We also noticed that the aging dynamics measured by SISF in MD simulations and stress decay in stress-relaxation experiments is more related to the enthalpy-relaxation revealed by sub-*T*_*g*_ annealing^32^. It has been widely accepted that enthalpy-relaxation is intrinsic to almost all glassy materials and are critical to some important scientific issues including physical aging, relaxation, and mechanical properties^33^. However, because few data points can be obtained in sub-*T*_*g*_ annealing experiments, the fitted *β* values may not be accurate, compared to that obtained in stress-relaxation experiments. Further systematic measurement of sub-*T*_*g*_ annealing may be critical to obtain convincing results, which will bring better understanding of glassy dynamics.

The secondary relaxation is generally believed to be the major relaxation dynamic of glasses below *T*_g_, when the *α*-relaxation is fully arrested. In both the MD simulations and stress-relaxation experiments, the target temperatures fall in the regions of secondary relaxation. In this sense, the aging dynamics of *β* = 3/7 observed in our work could be a property of the secondary relaxation. However, we also notice that the observed aging dynamics of $\beta=3/7$ may be independent of the secondary relaxation in MGs. Previous studies have shown that La_60_Ni_15_Al_25_, Pd_81_Si_19_, and Zr_50_Cu_40_Al_10_ MGs exhibit different secondary relaxation features below *T*_*g*_, which is also confirmed by our DMA measurements (see Supplementary Fig. 5a, c, e). However, they all exhibit the same stretching exponent $\beta=3/7$ in stress-relaxation below *T*_*g*_ (see Supplementary Fig. 5b, d, f). This indicates that the observed aging dynamics with $\beta=3/7$ could be intrinsic in MGs, independent of the intensity of the secondary relaxation. The correlation between the aging dynamics of *β* = 3/7 and secondary relaxation is worth exploring in future studies.

We also compared the aging behavior obtained in regular annealing and in strain-accelerated case in MD simulations. Since the dynamics at temperatures below *T*_*g*_ is too slow to be measured, we compared the SISFs of Zr_70_Cu_30_ MG at 700 K obtained in regular annealing and vibration-accelerated process, respectively (see Supplementary Fig. 9a). The applied sinusoidal strain does accelerate the aging process, compared to that in regular annealing. However, both decay curves show similar shape, and can be well-fitted by KWW function with $\beta=3/5$. We believe that this is also the case in the aging dynamics below *T*_*g*_. This indicates that the observed aging dynamics with $\beta=3/7$ below *T*_*g*_ is intrinsic, independent of vibration-accelerated process. We also compared the aging of Zr_70_Cu_30_ MG at 300 K under the static and vibrational strain (see Supplementary Fig. 9b). The decay curves of potential energy in Zr_70_Cu_30_ MG at 300 K under static and vibrational strain show similar behavior, and both curves can be well fitted by KWW function with $\beta=3/7$, indicating that the vibrational strain accelerates aging process, without changing its nature. Regarding the connection between MD simulation and the stress-relaxation experiment, we would like to emphasize that they both explore the same physical aging process. While it is much easier to calculate SISF in MD simulations, the stress relaxation can be measured much more easily in experiments. The difference in timescale might be attributed to the higher chances to exchange energies with the boundaries so the system can evolve toward equilibrium faster in MD simulation^34^.

From different aspects, we portray the aging dynamics of MG at room temperature as a unique one that differs from that above glass transition temperature. At a temperature well below *T*_g_, viscous flow is suppressed, whereas an enthalpy-driven aging dynamics reveals itself. In such aging process, through the non-affine displacements of a limited number of atoms, the total cavity of the system decays, leading towards a state with lower energy, while exhibiting a stretched exponential decay with characteristic stretching exponent $\beta=3/7$. In practice, the better understanding of room temperature aging dynamics of MGs helps to complete the puzzle of glassy dynamics, and provides theoretical guidance that helps in the prediction and tuning of aging of MGs.

## Methods

### MD simulations of vibration-accelerated aging

Classical MD simulations were performed using LAMMPS software^35^ for binary alloys of Zr_70_Cu_30_ and Zr_36_Cu_64_^21^, ternary alloy of La_60_Ni_15_Al_25_^22^, and monoatomic Ta^23^ systems with the interatomic interactions described by the realistic embedded-atom potentials. Each sample contains 40000 atoms in a cubic box with periodic boundary conditions applied in 3 directions. The time step was 2 fs. All samples were first equilibrated at temperature well above melting point for each system, then cooled to 300 K with cooling rate of 5 K/ps, during which system sizes were adjusted to give zero pressure in NPT ensemble. After relaxed for 1 ns at each temperature of interest, the ensemble was switched to NVT ensemble and the vibration-accelerated aging technique was applied to study aging dynamics in MGs at low temperatures. In this technique, a sinusoidal tension-compression strain of $\varepsilon={\varepsilon }_{0}{{\sin }}\left(2\pi t/{t}_{\omega }\right)$ was applied to MG samples along one direction, where ${\varepsilon }_{0}$ and ${t}_{\omega }$ are the applied maximum strain (vibrational amplitude) within the elastic limit and the period/frequency, respectively. This was inspired by MD-DMA (dynamical mechanical analysis) method, which mimics the experimental DMA in MD simulations^36,37^, but with a much longer simulation time so that the aging dynamics in MGs at low temperatures could be revealed. In our work, samples at room temperature (300 K) and around *T*_*g*_, i.e., 500 K (below *T*_*g*_), and 700 K (above *T*_*g*_) were chosen, so that the aging dynamics in glassy and supercooled liquid states can be compared.

### Self-intermediated scattering function

The relaxation dynamics was characterized by means of the self-intermediate scattering function (SISF),

1 $${F}_{s}\left(q,t\right)=\frac{1}{N}\mathop{\sum }\limits_{j=1}^{N}\left\langle {{\exp }}\left[-i{{{{{\bf{q}}}}}}\cdot \left({{{{{{\boldsymbol{r}}}}}}}_{j}\left(t\right)-{{{{{{\boldsymbol{r}}}}}}}_{j}\left(0\right)\right)\right]\right\rangle$$

where *q* is the wave-vector, *N* is the number of particles considered, $\left\langle .\right\rangle$ denotes thermal average, and ${{{{{{\boldsymbol{r}}}}}}}_{j}\left(t\right)$ is the position of particle *j* at time *t*. The scattering vector $q\, \approx \, 2.55{\mathring{\rm A} }^{-1}$ is chosen to be the first peak position of the structure factor measured at corresponding temperatures^4^. In the temperature range we were interested, 300 K~700 K, the first peak positions were almost the same, so that the same *q* value was used in the calculation of SISFs in the range of 300 K~700 K. That is, the choice of *q* value did not affect *β* value.

### Stress-relaxation experiments

#### Sample preparation

Master alloy ingots with the nominal atomic present compositions of Zr_70_Cu_30_, Zr_50_Cu_40_Al_10_, Zr_52.5_Cu_17.9_Al_10_Ni_14.6_Ti_5_ (Vit105), Zr_44_Ti_11_Cu_10_Ni_10_Be_25_ (Vit1b), La_60_Ni_15_Al_25_, Pd_81_Si_19_, Pd_52_Ni_32_P_16_ were prepared by arc-melting pure elements in argon atmosphere. The ingots were then fabricated into thin ribbons with a thickness of 20-35 µm by melt-spinning in argon atmosphere. The amorphous nature of MG ribbons was examined by X-ray diffraction (XRD) in a Bruker D8 AA25 diffractometer with Cu *Kα* radiation. *T*_g_ was determined by differential scanning calorimetry at a heating rate of 20 K/min in a Perkin-Elmer DSC 8000. Considering the enthalpy-driven nature of aging at room temperature, hyperquenched glassy ribbon samples, prepared by melt-spinning, other than well-relaxed ones were used for better measurements.

#### Stress-relaxation measurement

The stress-relaxation experiments were performed in a DMAQ800 TA instrument. After reaching target temperatures, the samples were stabilized for 30 minutes to relieve the artefacts in the stress measurement^38^. After stabilization process, a constant strain of 0.5% was applied on the ribbon and the stress evolution was simultaneously measured. After the stress-relaxation measurements, the samples are checked to verify the amorphous structure by XRD.

### Characterization of cavity in metallic glasses

To characterize the cavities in MD-generated metallic glassy samples, the numerical algorithms proposed by Sastry et al*.* was adopted^24^, in which an ‘exclusion sphere’ was defined around each atom. According to previous studies^39,40^, the exclusion radii were chosen to be 1.4 times atomic radii of Cu (1.27 Å) and Zr (1.58 Å), respectively, which is approximately the distance where the value of $g(r)$ starts to be nonzero^24^. In the calculation of cavity, Voronoi tessellation, weighted by the atomic radii of Zr and Cu, were firstly constructed, after which Delaunay tessellation was constructed to decompose the atomic structures into Delaunay simplexes. Subsequently, a single cavity was divided into a few neighboring Delaunay simplexes. The volume of each part of the cavity can be calculated by subtracting the volume occupied by atoms and the ‘exclusion region’ from the volume of the corresponding Delaunay simplex. Finally, each single cavity volume can be obtained by summarizing the volumes of all parts^24^. Cavity analysis was also performed with the choice of 1.3 times radius for the ‘exclusion sphere’, which shows similar decay characteristics (see Supplementary Fig. 10 for more detail).

## Supplementary information

Supplementary Information

## Data Availability

All relevant data that support the findings are available within this article and supplementary information and are also available from corresponding authors upon request.
