# Supplementary material for: Distinct relaxation mechanism at room temperature in metallic glass
Source: Nat Commun. 2023 Feb 1;14:540. doi: 10.1038/s41467-023-36300-x (PMC9892575; doi:10.1038/s41467-023-36300-x)
Supplement: Supplementary file 1 — Supplementary Information [file 41467_2023_36300_MOESM1_ESM.pdf]

Supplementary Information for
Distinct relaxation mechanism at room temperature in metallic glass

Yi-Tao Sun[1,#,*], Rui Zhao[1,2,#], Da-Wei Ding[1,3], Yan-Hui Liu[1,2,3], Hai-Yang Bai[1,2], Mao-Zhi Li[4,*], Wei-Hua Wang[1,2,3]

[1]Institute of Physics, Chinese Academy of Sciences, 100190 Beijing, China

[2]Center of Materials Science and Optoelectronics Engineering, University of Chinese Academy of Sciences, Beijing 100049, China

[3]Songshan Lake Materials Laboratory, Dongguan, Guangdong 523808, China

[4]Department of Physics, Beijing Key Laboratory of Opto-electronic Functional Materials & Micro-nano Devices, Renmin University of China, Beijing 100872, China

[#]These authors contributed equally: Yi-Tao Sun, Rui Zhao

*e-mail: sunyitao@iphy.ac.cn; maozhili@ruc.edu.cn

Supplementary Note 1, The effects of amplitude and frequency of the applied sinusoidal strain

Supplementary Note 2, Aging with different waiting time

Supplementary Note 3, Stress-relaxation experimental data

Supplementary Note 4, Radial distribution function and Vorornoi polyhedral analysis

Supplementary Note 5, Comparison between regular aging and accelerated aging

Supplementary Note 6, Cavity analysis

Supplementary Note 1, The effects of amplitude and frequency of the applied sinusoidal strain

The effects of amplitude and frequency of the applied sinusoidal strain are shown in Fig. 1 below. It is clear that the effect of period (5 - 50 ps) is insignificant to the evolution of both potential energy and SISF, while the value of strain amplitude (0.01 - 0.03) has significant effect on the rate of decay for potential energy and SISF. All data can be well fitted by KWW function with $\beta = 3/7$, as shown in Fig. 1. The increase of strain amplitude (within elastic limit) brings the system towards a lower energy state without changing the dynamical signature of $\beta = 3/7$.

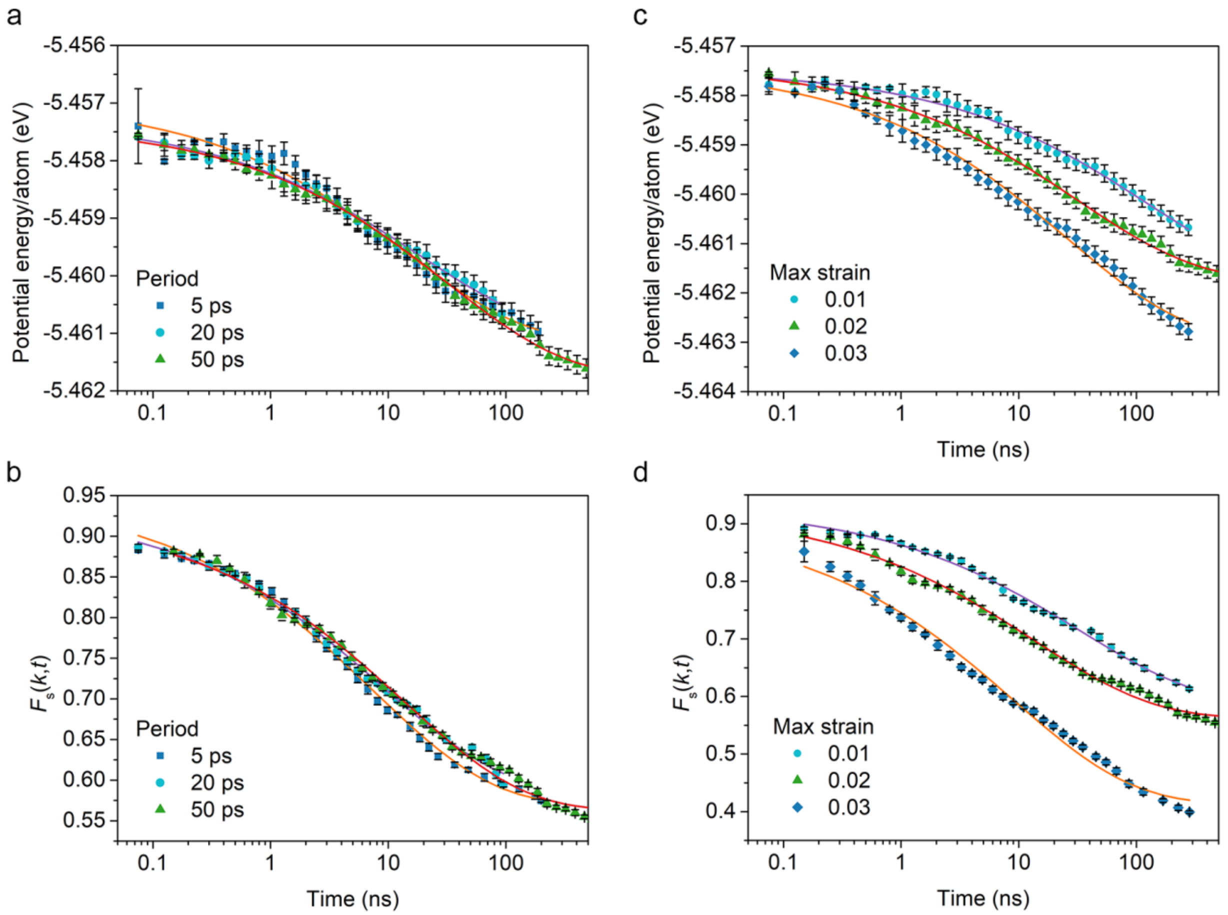

Supplementary Fig. 1 | The effects of amplitude and frequency of the applied sinusoidal strain. Decay curves of potential energy (a,c) and SISF (b,d) of $Zr_{70}Cu_{30}$ MGs in accelerated aging at 300 K with different periods and strain amplitudes, respectively. Results are fitted by KWW function with $\beta = 3/7$. Error bars represent standard deviations.

Supplementary Note 2, Aging with different waiting time

Decay curves of potential energy and SISF of $Zr_{70}Cu_{30}$ MGs in accelerated aging at 300 K, with different waiting time are shown in Fig. 2. Results are fitted without constraining the value of β. We can see that more relaxed glasses (longer waiting time) yield larger value of β and longer relaxation time.

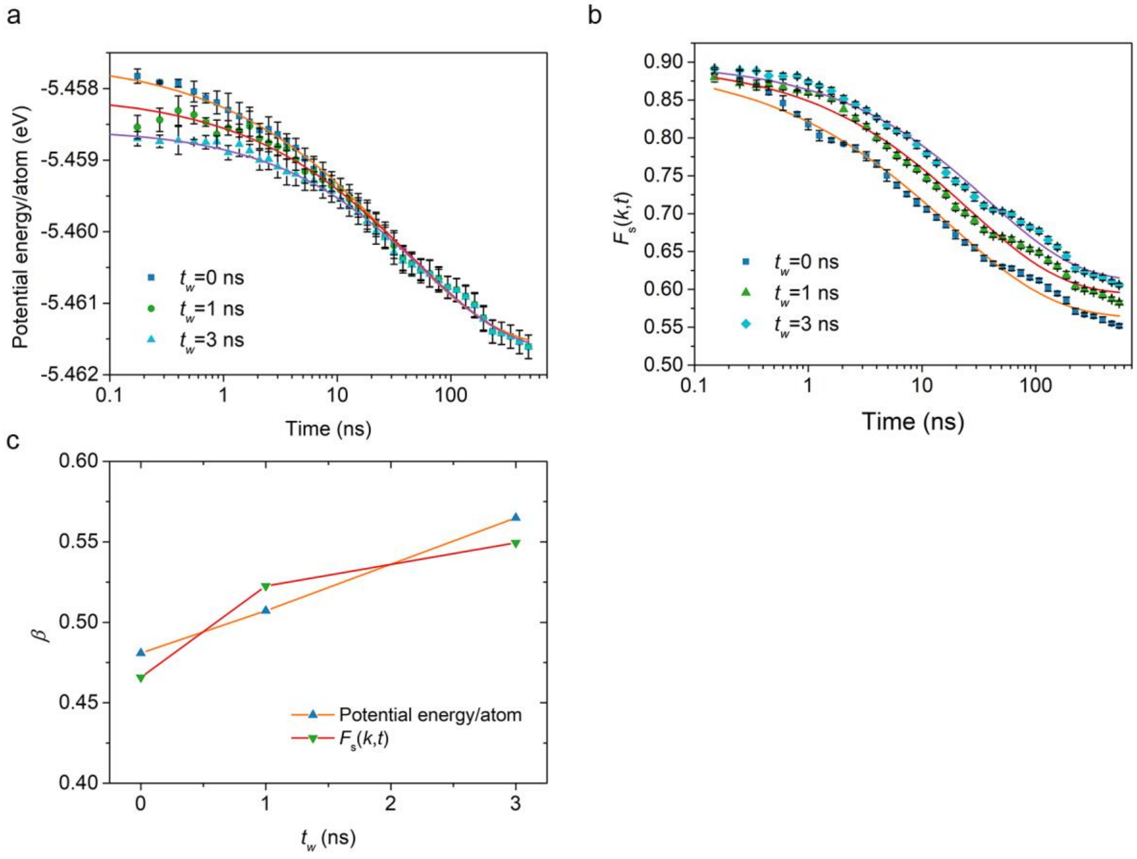

Supplementary Fig. 2 | Aging with different waiting time. Decay curves of potential energy (a) and SISF (b) of $Zr_{70}Cu_{30}$ MGs in accelerated aging at 300 K, with different waiting time. Results are fitted without constraining the value of β. (c) evolution of β value with waiting time. More relaxed glasses (longer waiting time) yield larger value of β and longer relaxation time. Error bars represent standard deviations.

We also did an analytical calculation to further elucidate this issue. Assuming a KWW function of $f(x) = \exp\left(-(x + t_0)^{3/7}\right)$, where $t_0 = \ln(y_0)^{7/3}$ is waiting time and y_0 represents the corresponding initial state, the decay behavior and fitting at various initial states of $y_0 = 1, 0.9, 0.8 \ldots 0.1$ are shown in Fig. 3. The value of β increases naturally with increasing waiting time, indicating the intrinsic property of stretching parameter on initial state.

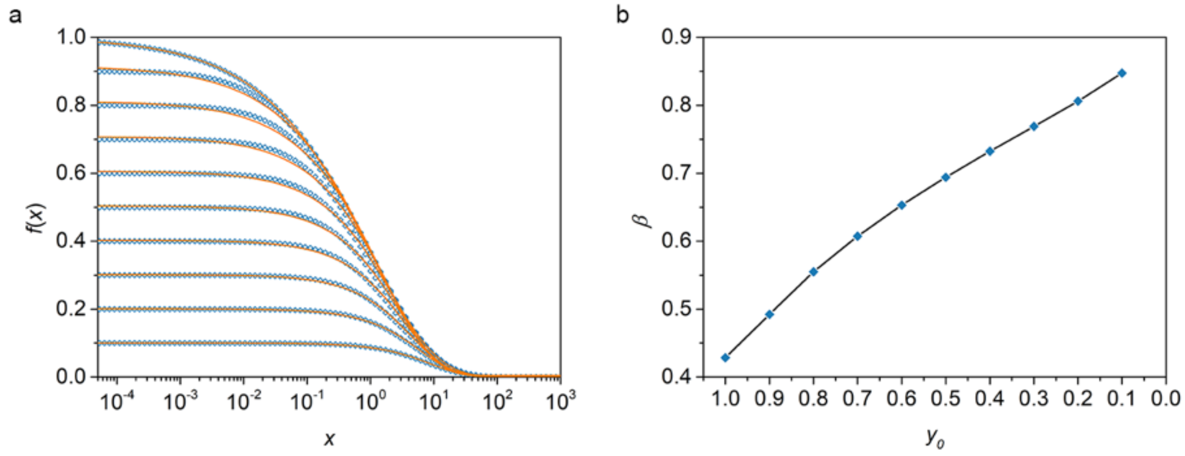

Supplementary Fig. 3 | Evolution of β for a standard KWW function with different initial states. (a) KWW fitting on a stretched exponential decay with β=3/7 and different waiting time. (b) The evolution of β with initial states.

Supplementary Note 3, Stress-relaxation experimental data

More data from stress-relaxation experiments are shown in Fig. 4 and 5. The loss modulus curves as a function of temperature were measured at a constant rate of 3 K/min with a testing frequency of 1 Hz and an amplitude of 10 μm.

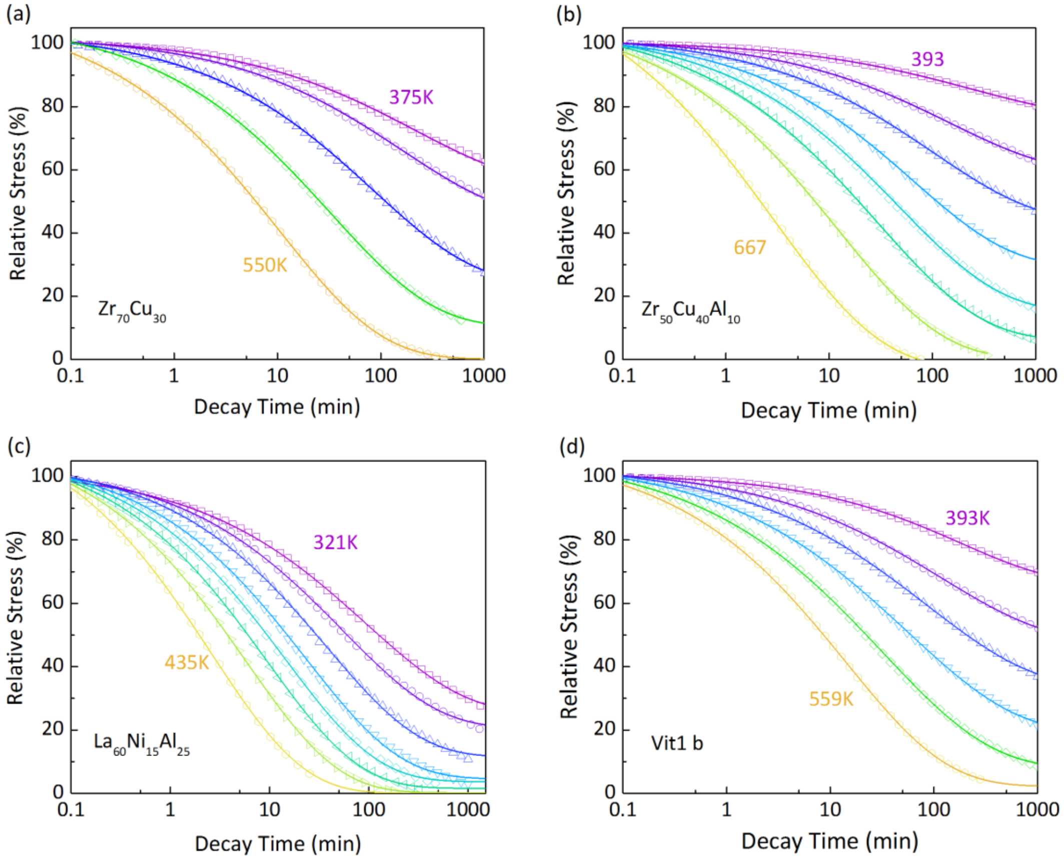

Supplementary Fig. 4 | Stress-relaxation curves of various metallic glasses. (a) Stress-relaxation curves of $Zr_{70}Cu_{30}$ ribbons at T=550, 500, 450, 400, 375 K (from left to right). (b) Stress-relaxation curves of $Zr_{50}Cu_{40}Al_{10}$ ribbons, T=667, 632, 593, 562, 527, 483, 443, 393 K. (c) Stress-relaxation curves of $La_{60}Ni_{15}Al_{25}$ ribbons, T=435, 423, 412, 400, 387, 366, 344, 321 K. (d) Stress-relaxation curves of Vit1 b ribbons, T=559, 528, 497, 466, 435, 393 K. All decay curves can be well fitted by KWW function. With decreasing temperature, the relaxation time and residual stress gradually increase. However, for different MG ribbons at the whole experimental temperature range, the fitted stretching exponent β values remain to be around 3/7.

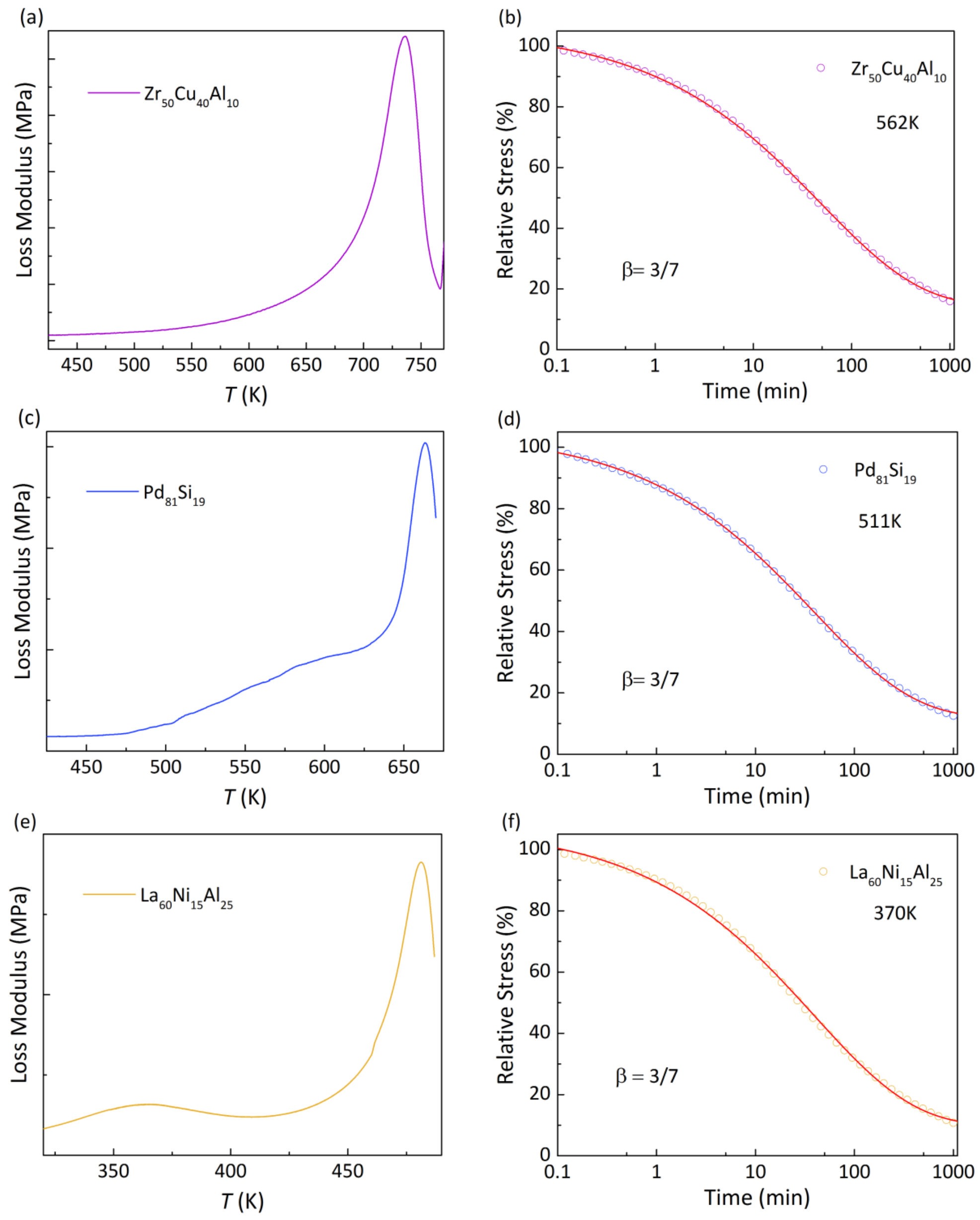

Supplementary Fig. 5 | Stress-relaxation for metallic glasses with different secondary relaxations. (a)(c)(e) Loss modulus profiles as a function of temperature of $Zr_{50}Cu_{40}Al_{10}$, $Pd_{81}Si_{19}$ and $La_{60}Ni_{15}Al_{25}$ ribbons, respectively. The secondary relaxation appears at low temperatures on the left side of α-relaxation peak, i.e., a long tail for $Zr_{50}Cu_{40}Al_{10}$, a hump for $Pd_{81}Si_{19}$, and a pronounced peak for $La_{60}Ni_{15}Al_{25}$, respectively. (b)(d)(f) Stress relaxation profile of $Zr_{50}Cu_{40}Al_{10}$, $Pd_{81}Si_{19}$ and $La_{60}Ni_{15}Al_{25}$ ribbons at 562K, 511K and 370K, respectively. Although these MGs exhibit distinct secondary relaxation behaviors, they show the same stress relaxation dynamics with β=3/7.

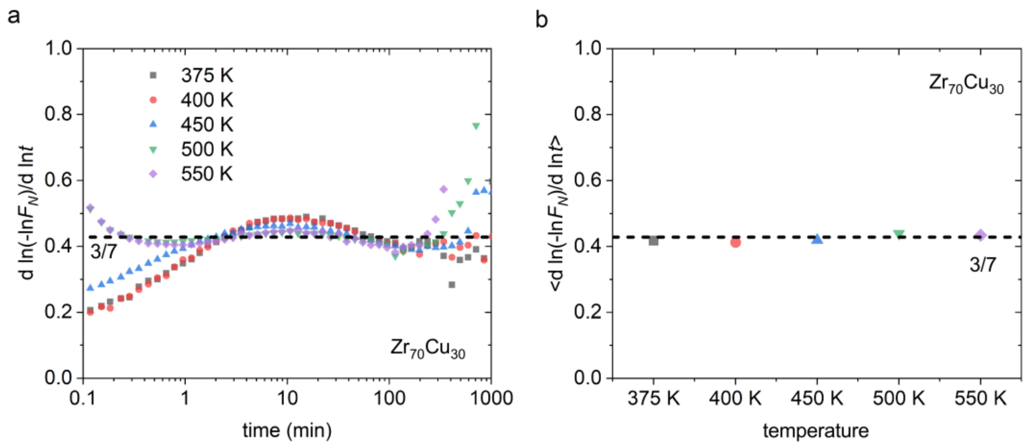

Supplementary Fig. 6 | Stretching exponent analysis for $Zr_{70}Cu_{30}$ at different temperatures. $\mathrm{d}\ln(-\ln F_N)/\mathrm{d}\ln t$ curves (a) and averaged $\mathrm{d}\ln(-\ln F_N)/\mathrm{d}\ln t$ (b) of $Zr_{70}Cu_{30}$ at different temperatures. The data almost collapse on the dashed line which marks the value of 3/7, further supporting our conclusion.

Supplementary Note 4, Radial distribution function and Voronoi polyhedral analysis

Radial distribution function (RDF) and Voronoi polyhedral analysis for $Zr_{70}Cu_{30}$ MG during accelerated aging does not show clear correlation with the decay of energy or SISF.

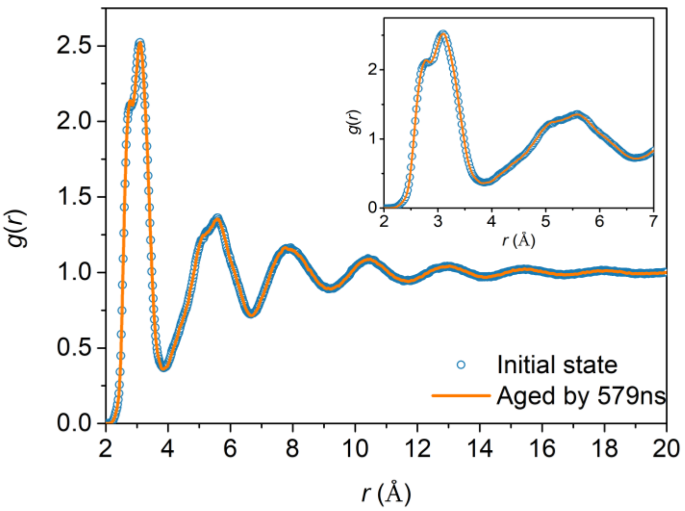

Supplementary Fig. 7 | Radial distribution function of $Zr_{70}Cu_{30}$ MG before aging and after 579 ns of accelerated aging at 300 K. Inset shows partial enlarged view for better clarity.

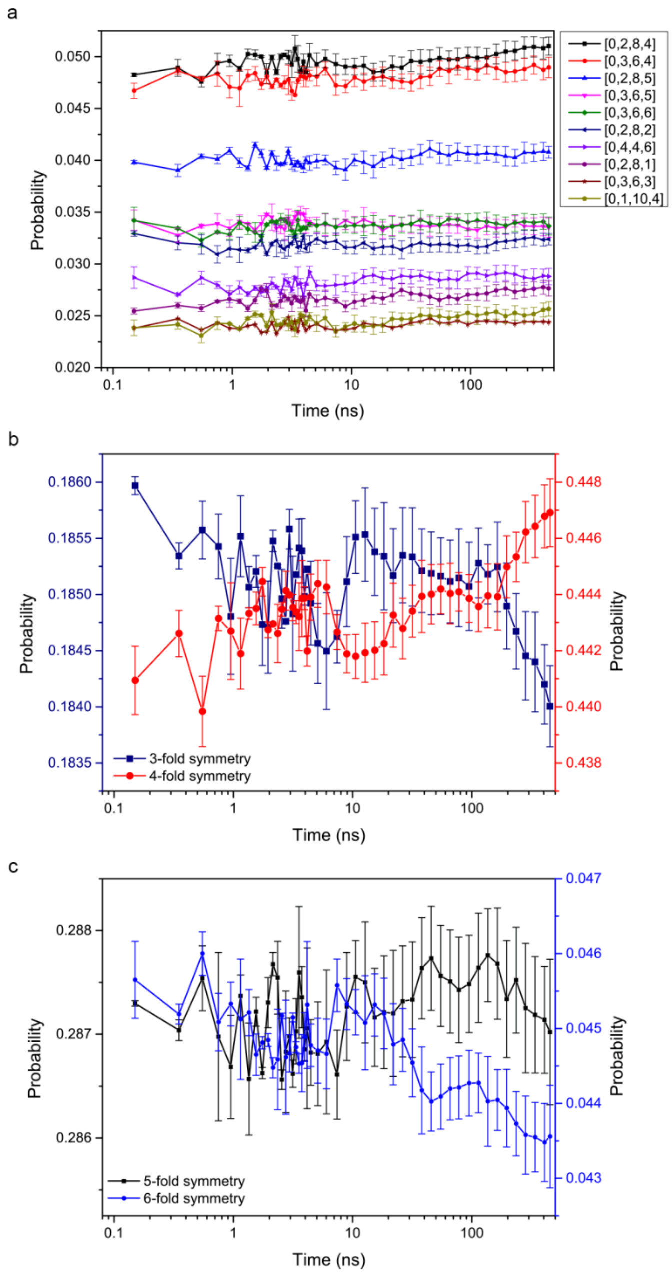

Supplementary Fig. 8 | Voronoi polyhedral analysis for $Zr_{70}Cu_{30}$ metallic glass during accelerated aging. (a) Probability evolution of the top 10 Voronoi polyhedral during aging. (b) Probability evolution of the 3 and 4 -fold symmetry calculated from Voronoi polyhedral analysis during aging. (c) Probability evolution of the 5 and 6 -fold symmetry calculated from Voronoi polyhedral analysis during aging. Error bars represent standard deviations.

Supplementary Note 5, Comparison between regular aging and accelerated aging

The SISFs of $Zr_{70}Cu_{30}$ MG in regular and accelerated aging at 700 K show similar shape of KWW decay, both being well fitted with $\beta = 3/5$. The potential energy evolution of $Zr_{70}Cu_{30}$ MG subjected to static strain of 0.02 and periodic strain (maximum strain of 0.02) at 300 K also show similar shape of KWW decay, and both can be well fitted with $\beta = 3/7$. In static strain, the potential energy is measured at strain of 0.02. In periodic strain, the potential energy is measured at strain of 0, see Fig.1a in the main text. Therefore, the potential energy is higher for sample in static strain.

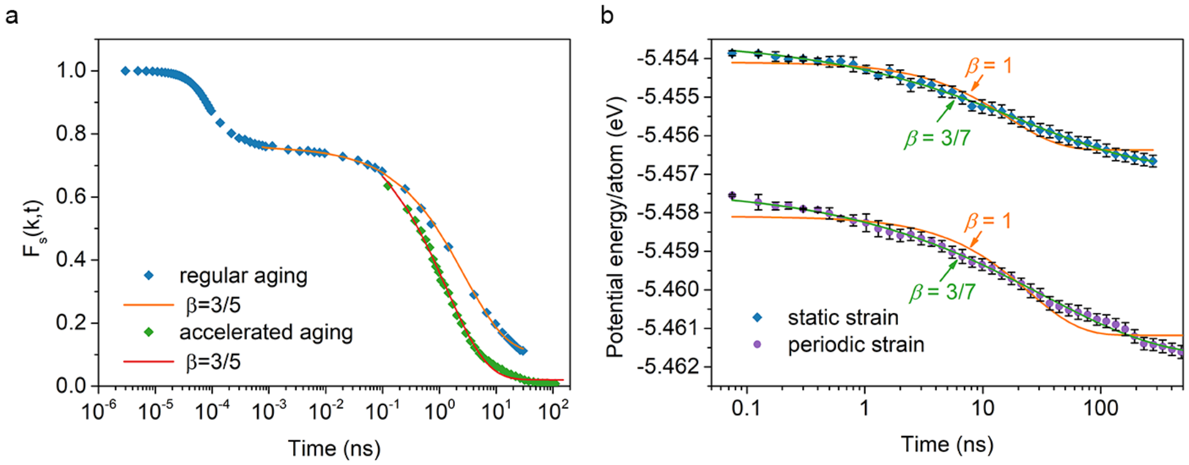

Supplementary Fig. 9 | Comparison between regular aging and accelerated aging. (a) SISFs calculated from regular aging and accelerated aging at 700 K, both being fitted with $\beta = 3/5$. (b) Potential energy evolution of $Zr_{70}Cu_{30}$ MGs subjected to static strain of 0.02 and periodic strain (strain amplitude of 0.02) at 300 K, both being well fitted with $\beta = 3/7$. Error bars represent standard deviations.

Supplementary Note 6, Cavity analysis

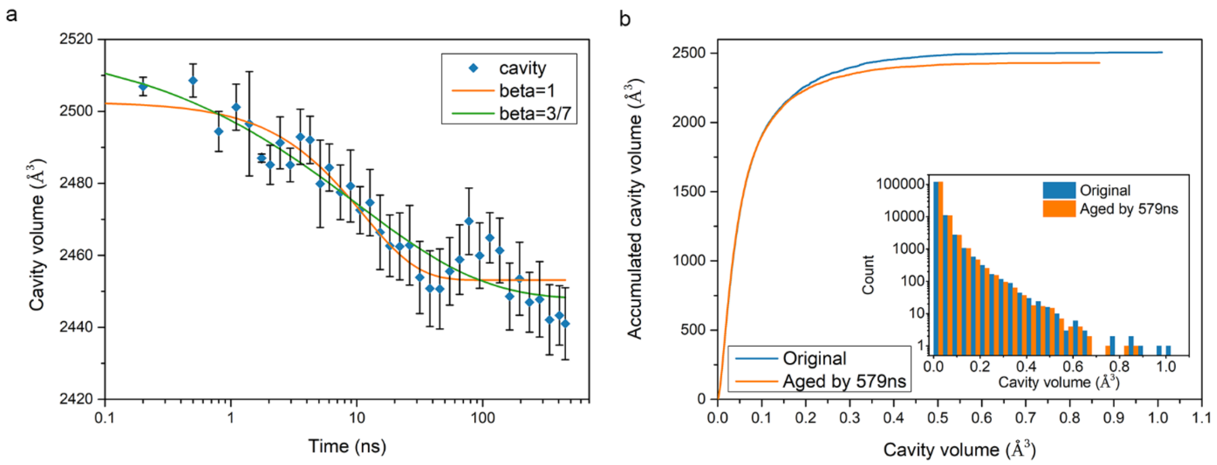

Supplementary Fig. 10 | Cavity analysis calculated using 1.3 times radius (instead of 1.4 times radius in the main text) for $Zr_{70}Cu_{30}$ MG during accelerated aging. (a) Total cavity volume decay during accelerated aging. The solid green line and orange line show fits of KWW function with stretching exponent $\beta = 3/7$ and $\beta = 1$, respectively. (b) Accumulated cavity volume with respect to cavity volume before and after aging. Inset shows volume distribution of cavities. Results shows a significance decrease of relatively larger cavities. Error bars represent standard deviations.
